# Pediatric Deep Brain Stimulation Using Awake Recording and Stimulation for Target Selection in an Inpatient Neuromodulation Monitoring Unit

**DOI:** 10.3390/brainsci8070135

**Published:** 2018-07-17

**Authors:** Terence D. Sanger, Mark Liker, Enrique Arguelles, Ruta Deshpande, Arash Maskooki, Diana Ferman, Aprille Tongol, Aaron Robison

**Affiliations:** 1 Department of Neurology, Children’s Hospital Los Angeles, Los Angeles, CA 90027, USA; dferman@usc.edu; 2 Department of Biomedical Engineering, Viterbi School of Engineering, University of Southern California, Los Angeles, CA 90089-1111, USA; enrique.arguelles@usc.edu (E.A.); rutadesh@usc.edu (R.D.); maskooki@usc.edu (A.M.); tongol@usc.edu (A.T.); 3 Department of Neurosurgery, Children’s Hospital Los Angeles, Los Angeles, CA 90027, USA; liker@usc.edu (M.L.); arobison@chla.usc.edu (A.R.)

**Keywords:** deep brain stimulation, secondary dystonia, pediatric, targeting, stereo EEG

## Abstract

Deep brain stimulation (DBS) for secondary (acquired, combined) dystonia does not reach the high degree of efficacy achieved in primary (genetic, isolated) dystonia. We hypothesize that this may be due to variability in the underlying injury, so that different children may require placement of electrodes in different regions of basal ganglia and thalamus. We describe a new targeting procedure in which temporary depth electrodes are placed at multiple possible targets in basal ganglia and thalamus, and probing for efficacy is performed using test stimulation and recording while children remain for one week in an inpatient Neuromodulation Monitoring Unit (NMU). Nine Children with severe secondary dystonia underwent the NMU targeting procedure. In all cases, 4 electrodes were implanted. We compared the results to 6 children who had previously had 4 electrodes implanted using standard intraoperative microelectrode targeting techniques. Results showed a significant benefit, with 80% of children with NMU targeting achieving greater than 5-point improvement on the Burke–Fahn–Marsden Dystonia Rating Scale (BFMDRS), compared with 50% of children using intraoperative targeting. NMU targeting improved BFMDRS by an average of 17.1 whereas intraoperative targeting improved by an average of 10.3. These preliminary results support the use of test stimulation and recording in a Neuromodulation Monitoring Unit (NMU) as a new technique with the potential to improve outcomes following DBS in children with secondary (acquired) dystonia. A larger sample size will be needed to confirm these results.

## 1. Introduction

Positive results of deep brain stimulation (DBS) in primary (genetic or isolated) dystonia [1,2,3,4] suggest the potential of this procedure for benefit in children with cerebral palsy (CP) [5] and other causes of secondary (acquired or combined) dystonia [6]. However, recent consensus meetings have highlighted that the benefits in secondary dystonia are much less pronounced and often not reflected in standard outcome measures [7], suggesting that while the procedure has promise in this population, it does not fully ameliorate the primary symptoms. The standard target for dystonia in children is the internal globus pallidus (GPi) [2], based on use of this target to reduce dyskinesias in patients with Parkinson’s disease. Earlier results had shown efficacy of thalamotomy to treat dystonia in this population [8,9,10], but thalamus has not been a significant recent target for DBS in children.

While there is some commonality of injury in children with secondary dystonia, often due to hypoxic-ischemic injury to basal ganglia and lateral thalamus [11,12], variation in response to medication, functional neurosurgery, and therapy suggests significant heterogeneity of the processes leading to dystonia. For this reason, we hypothesize that the optimal target for DBS is likely to vary between children, even when symptoms are similar.

Current techniques for DBS implantation typically involve either stereotactic surgery with target confirmation by microelectrode recording (MER), or intraoperative MRI-guided implantation [13,14]. When MER is performed, the patient is usually required to be awake. When patients are awake, test stimulation can be performed in order to ensure that there are no side effects that would limit postoperative stimulation options. Awake procedures can be problematic in children with dystonia because of the risk that dystonia will cause involuntary movement while the child is in a head frame on the operating table. MRI-guided implantation is performed under general anesthesia and avoids this risk, but it does not allow recording or stimulation and thus requires that the correct target can be identified based only on MRI anatomical imaging [15]. The procedure we describe here avoids the risks of awakening children in the operating room while permitting safe microelectrode recording and test stimulation.

The situation facing DBS in secondary dystonia is similar to resection surgery for refractory epilepsy, where the epileptogenic focus is different for different children and thus needs to be precisely localized prior to resection [16]. Unlike resection, DBS is a reversible procedure, yet precise target localization is nonetheless critical to efficacy. We describe a new procedure based on the use of stereo EEG depth electrode technology that is commonly used for identification of epileptic foci. We propose to use a similar technique for identification of targets for deep brain stimulation. In brief, temporary depth electrodes are used to test stimulation and recording at multiple possible targets. This information is then used for subsequent implantation of permanent DBS electrodes. We report on the first 9 cases of children undergoing the new procedure, and we compare to 6 children implanted using standard intraoperative microelectrode recording and stimulation.

## 2. Materials and Methods

### 2.1. Patient Selection

Criteria for surgical candidacy at our institution include: (1) presence of dystonia that results in significant limitation of function or interference with care, (2) identifiable potential stimulation targets on MRI, (3) failure of symptomatic (or when known, etiologic) medical therapy. Low level of cognitive function or the presence of other movement disorders are not exclusion criteria. Dystonia is diagnosed by a pediatric movement disorder specialist (T.D.S.) based on published criteria [17,18]. Confirmation of failure of medical therapy requires adequate dosing and duration of potentially effective oral medications, usually including (1) levodopa/carbidopa (at least 2 weeks at 10 mg/kg/day), (2) trihexyphenidyl (at least 2 months at 0.75 mg/kg/day), and (3) baclofen (at least 1 month at 0.5 mg/kg/day) [19]. Escalation rate, maximum dose, duration, and concomitant medications need to be adjusted carefully to ensure that failure of medical therapy is confirmed by an adequate medication trial. All patients undergo informed consent for surgical procedures in accordance with standard hospital practice. Patients or parents of minor patients also sign informed consent for research use of electrophysiological data (University of Southern California Human Subjects Institutional Review Board approval UP-13-00521, 15 November 2013 to 12 September 2018) and HIPAA authorization for research use of protected health information.

### 2.2. First-Phase Surgery

Potential targets for stimulation are identified by consultation with Department of Neurology and Department of Neurosurgery. Candidate targets are based on reports in the literature of efficacy in dystonia through either lesions or stimulation in thalamus and basal ganglia. Targets may include internal globus pallidus (GPi), subthalamic nucleus (STN), and subnuclei of the thalamus, identified using Hassler’s terminology as VA, Voa/Vop, Vim, and VPL [20]. Voa/Vop are the output projection areas from GPi, Vim is the projection area from cerebellar nuclei, and VPL is the projection area from the spinothalamic and lemniscal sensory systems. For conciseness we will refer to Voa/Vop as “Vo”.

Each child has a preoperative anatomic scan performed with parameters determined by requirements for the “Stealth(tm)” stereotaxy software (Medtronic Inc., Minneapolis, MN, USA). A Radionics CRW head frame (Integra Life Sciences Corp, Burlington, MA) is attached under general endotracheal anesthesia, and CT scan performed. The CT scan is co-registered to the preoperative MRI, and stimulation targets on MRI are referenced to the frame coordinates using the Stealth(tm) software. Up to 10 Adtech MM16C depth electrodes (Adtech Medical Instrument Corp., Oak Creek, WI, USA) are placed through drill-holes using frame guidance. Electrodes are fixed to the skull using 13 mm anchor bolts (Adtech LSBK1-BX-06, Adtech Medical Instrument Corp.). The frame is removed and a postoperative CT scan is performed to confirm location and ensure lack of hemorrhage or other complications.

### 2.3. NMU Testing Protocol

In order to permit full recovery from the effects of general anesthesia, testing does not start until at least 24 h after surgery. Testing includes three categories of data: (1) stimulation, (2) recording, (3) evoked potentials. MM16C electrodes have a diameter of 1.2 mm, and each contains 6 low-impedance contacts (1–2 kOhm) that run circumferentially around the electrode in a 2 mm band, with 5 mm distance between the centers of each band. Each electrode also contains 10 high-impedance contacts (70–90 kOhm) that are approximately 500 µm circular and arranged in groups of 2 or 3 circumferentially around the electrode and between the low-impedance electrode bands. The high impedance contacts protrude slightly from the surface of the electrode. Low-impedance contacts can be used for stimulation and for recording of local field potentials (LFP). High-impedance contacts can be used for recording single-unit activity and for high-frequency components of the LFP. Video is recorded continuously and synchronized to the intracranial recordings using either an in-room camera or via USB-connected peripheral camera. Surface electromyography (EMG) is recorded using up to 16 Delsys DE2.1(tm) or Delsys Trigno(tm) electrodes (Delsys Corp., Natick, MA, USA) attached over the belly of the biceps, triceps, wrist flexor and extensor groups, quadriceps, medial hamstrings, tibialis anterior, and medial gastrocnemius muscles.

Intracranial stimulation is performed using a Medtronic model 37022 external neurostimulator (Medtronic Inc.) controlled by an 8840 programmer. After confirmation of impedance, stimulation is performed through the low-impedance contacts at bilateral (left and right-sided) contacts with settings of 90 µs 60 or 185 Hz, and increasing voltage up to 5 V, for a total of 3–5 min per condition. Stimulation is always bipolar at adjacent contacts, with the more distal contact negative and the more proximal contact positive. To perform bilateral stimulation, two neurostimulators and two programmers are used. Testing all electrode pairs typically requires 4 to 6 h, depending on the cooperation level of the patient.

Recording is performed using either a NeuroOmega(tm) 96-channel system (AlphaOmega Co USA Inc., Alpharetta, GA, USA) or a PZ5M 256-channel digitizer connected to an RZ2 processor and RS4 high speed data storage (TDT, Tucker-Davis Technologies Inc., Alachua, FL, USA). Intracranial electrodes are connected through Adtech Cabrio(tm) connectors via shielded cables to inputs on each amplifier. Low impedance contacts are recorded at 1.375 kHz (Alpha Omega) or 2 kHz (TDT) sampling rate with digital bandpass filtering between 2 and 400 Hz. High impedance contacts are recorded at 22 kHz with bandpass filtering between 300 Hz and 9 kHz. Low impedance recordings are referenced to a ground electrode placed on the neck or back, while high impedance recordings are referenced separately within each electrode to the most proximal low-impedance contact. Single unit activity is detected on the high impedance contacts by level crossings, and spike sorting is performed on recorded data using clustering in the space of principal components. Recording during active tasks typically requires 3 to 4 h. Recording is continued throughout the hospitalization, so that data are available while patients are active, awake resting and asleep.

Evoked potentials are recorded during stimulation of the median nerve at the wrist or the tibial nerve at the popliteal fossa. Stimulation is performed using an XLTech NeuroMax 1002 clinical EMG stimulator (Natus Medical Inc., Pleasanton, CA, USA) at 9 Hz, 500 µs, and current increasing to just above sensory threshold (typically 8–12 mA), with stimulus-locked potentials recorded and averaged from both low impedance and high impedance contacts. Evoked potentials are calculated offline by stimulus-triggered averaging, with the trigger determined by level crossing of the stimulus artifact. Testing requires approximately 30 min.

### 2.4. Testing Outcomes

During stimulation, the patient is observed for beneficial effects, which usually requires assessment of a specific task such as writing, self-feeding, speaking, reaching, sitting, or walking, depending on the child’s abilities and the parent’s goals. In our experience, any results of stimulation in thalamus are visible within a few seconds, whereas results of stimulation in globus pallidus can sometimes take up to several weeks and thus we do not always see benefits from GPi stimulation during the first phase hospitalization.

We also observe for side effects of stimulation, including paresthesias, discomfort, worsening dystonia, visual scotomata, or other complaints. Side effects that occur at higher voltages (above 4.5 V) are less concerning than effects at lower voltages that would limit subsequent clinical stimulation.

**Figure 1:**
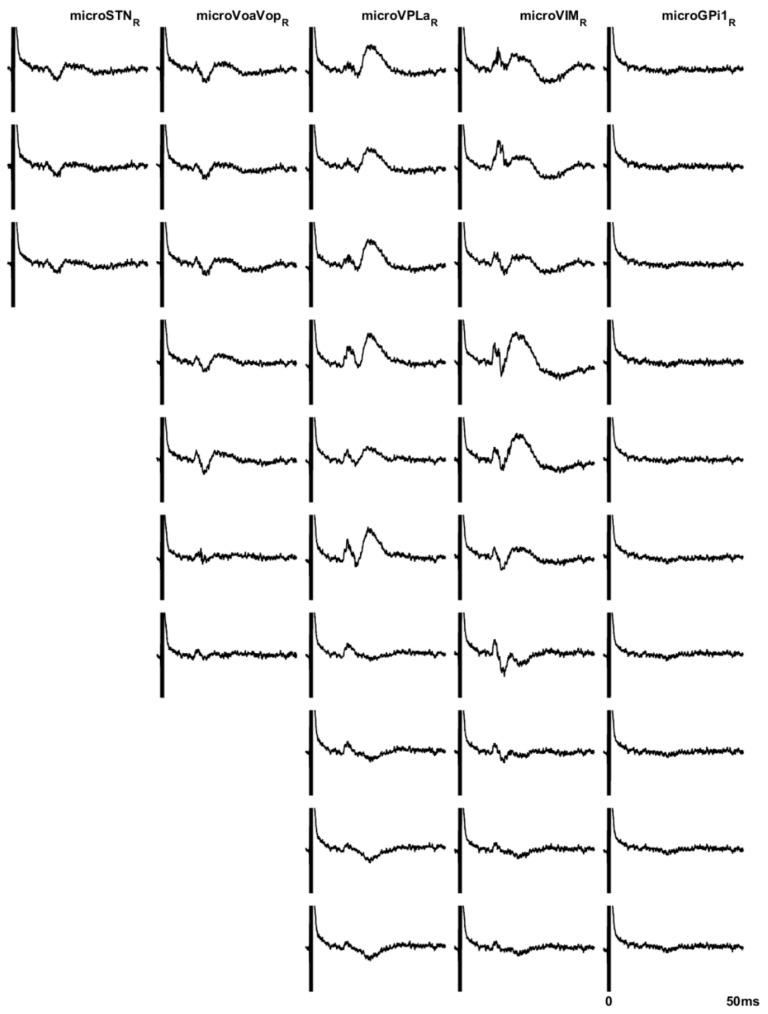
Example of evoked potentials recorded on the high-impedance contacts in (from left to right) subthalamic nucleus (STN), VoaVop, VPL, Vim, and globus pallidus (GPi). Top to bottom traces show averaged data from proximal to distal contacts. Vim and VPL have a robust response at approximately 14 ms after the stimulus, whereas a much smaller response is seen in Vo and STN.

Evoked potential testing is helpful for identifying thalamic sub-nuclei [21]. Vim and VPL nuclei respond to peripheral stimulation, whereas Voa/Vop and VA do not [22]. Therefore, the presence of an evoked potential provides physiological evidence to support electrode positioning in either Vim or VPL, and a difference in response between two adjacent electrodes can be used to identify the Vop/Vim boundary. This is particularly important because the standard atlas identification of thalamic nuclei may not be fully applicable in children, particularly in children with brain injury for whom there may be atrophy or remapping of thalamus. Figure 1 shows an example of evoked potentials recorded on the high impedance channels.

**Figure 2:**
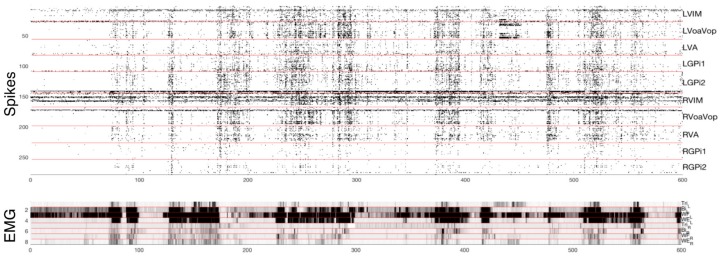
Top: microelectrode recordings with identified spikes from 250 distinct cells. Intracerebral recording locations are identified on the right. Bottom: Muscle activity (electromyograph, EMG) for 8 muscles with higher level of EMG plotted as dark. From top to bottom, muscles are Left triceps, biceps, wrist flexor group, wrist extensor group, right triceps, biceps, wrist flexor group, wrist extensor group. Horizontal axis is in seconds, and a total of 10 min are shown. Data are from patient NMU8 during attempted left arm movement.

Single unit recordings are correlated against dystonic contractions seen on EMG and video. Although it is not known whether single unit activity predicts DBS efficacy, we assume that regions for which there is no correlation with dystonic contractions are unlikely to be a cause of those contractions and therefore less likely to be effective targets for stimulation. The use of the single-unit data is particularly helpful for assessing targets within GPi, since stimulation in GPi does not always produce immediate effects and is therefore less useful for predicting future effects of stimulation. Figure 2 shows a sample of simultaneous single-cell spike data from 250 identified cells, and the corresponding muscle activity during attempts at left arm movement. In this case, right Vo appears to be correlated best with left-sided dystonic arm movement, whereas involuntary overflow to the right-sided muscles correlates with multiple brain regions but most prominently with left Vo.

### 2.5. Target Selection

When the testing phase is complete (usually requiring 4–6 days total, depending on stimulation effects and patient cooperation with testing) a post-testing CT scan is performed to confirm that leads have not moved, and the leads are then removed at the bedside. 10–14 days later, permanent leads (Medtronic 3387) are implanted at the location of the 4 electrodes predicted to be most effective. Criteria for target selection include: (1) improvement in function during stimulation at macro-contacts, (2) lack of side effects during stimulation at macro-contacts up to 5 V, (3) single-cell firing that either increases or decreases when dystonia is present.

**Figure 3:**
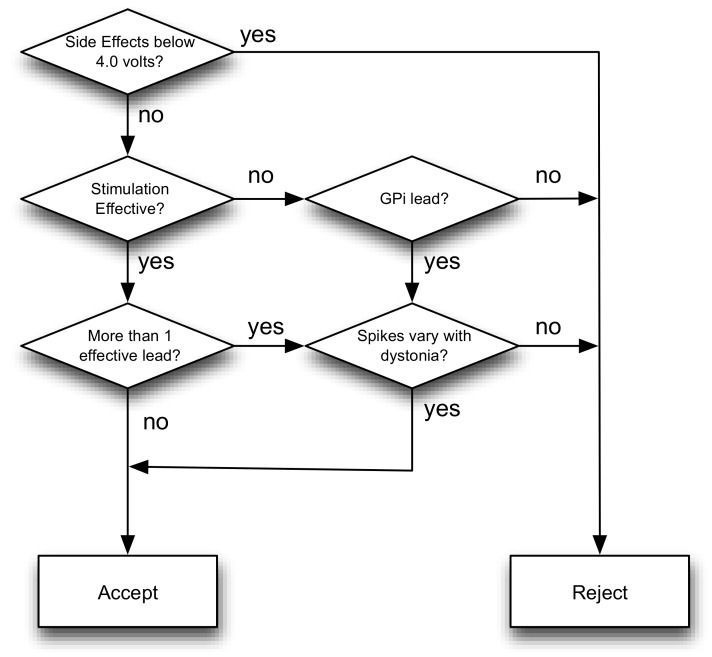
Simplified flowchart of decision-making process based on NMU data. When more than 1 lead is effective during stimulation, we select the lead for which the single-cell spikes correlate most closely with dystonic muscle activity.

While stimulation in thalamus typically produces beneficial effects (if any) within a few seconds, we do not always obtain such rapid changes from stimulation in GPi. In the absence of side effects, we always implant GPi based on previous clinical experience suggesting that this it is an effective target in many forms of dystonia [5,14]. When two different potential targets within GPi are tested, we select the one with the single-cell firing most correlated with dystonic EMG. When two different thalamic targets appear to be equally effective without side effects, we again choose based on single-cell firing. Figure 3 provides a simplified decision flowchart, but we emphasize that decision-making needs to be individualized to the needs and responses of individual patients.

If evoked potential testing suggests that leads are placed asymmetrically (for example, if the Vo lead on one side has a robust evoked potential then that lead is more likely too posterior, responding to activity in Vim), then locations of the permanent stimulating electrodes are corrected to achieve predicted symmetric locations as close as possible to effective stimulation points.

### 2.6. Second-Phase Surgery

**Figure 4:**
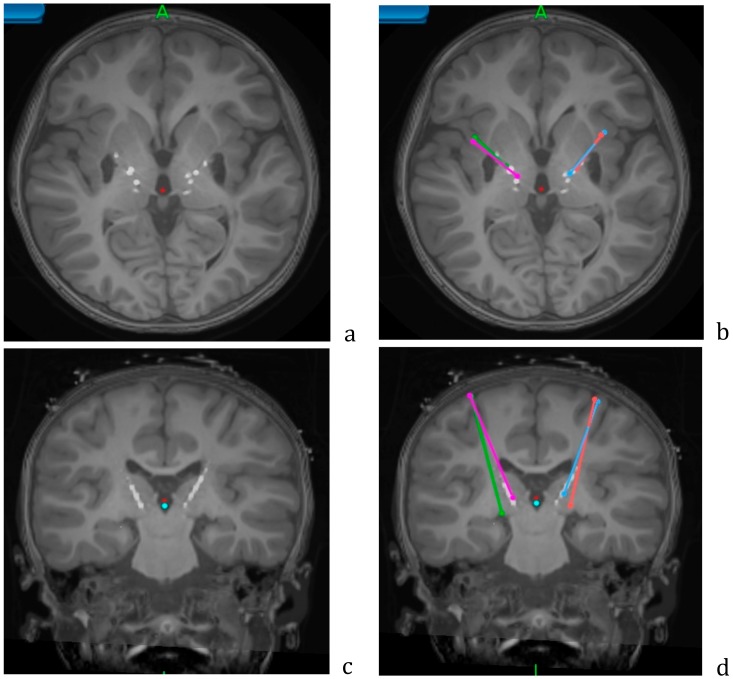
Axial (**a**,**b**) and coronal (**c**,**d**) views of the postoperative CT overlaid on the preoperative MRI, showing the lead locations for the Adtech stereo EEG leads. Planned trajectories for permanent leads are shown in color (**b**,**d**). Data from patient NMU4.

Implantation is performed under general anesthesia using procedures identical to those for the first-phase surgery. Targeting is determined by fusing the pre-operative MRI and post-operative first-phase CT to the second-phase CT performed with the CRW frame in place. When 4 electrodes are implanted, the entry point is adjusted so that electrodes on the same side can be placed through the same burr-hole and fixed by surgical modification of the Stimloc(tm) plastic cap. Electrodes are buried beneath the skin. Figure 4 shows an example of targeting using the Medtronic StealthStation(tm) system in patient NMU4 (see Table 1).

### 2.7. Third-Phase Surgery

A total of 10–14 days after the second-phase surgery, extension leads (Medtronic 7483) are tunneled from the buried ends of the intracranial electrodes to implanted stimulators (Medtronic Activa RC or PC) placed in the chest. In order to facilitate programming with differing frequencies in different targets, homologous leads are routed to the same stimulator, typically with both GPi leads connected to the stimulator in the left chest, and both thalamic (or other target) leads connected to the stimulator in the right chest.

### 2.8. Peri-Operative Medication

Anesthesia for all surgeries is performed with generalized endotracheal gas anesthesia, typically sevoflurane. Postoperative pain is readily controlled with acetaminophen.

Preferred antibiotics include intravenous vancomycin and ceftazidime perioperatively during first-phase surgery. For second-phase surgery, intravenous vancomycin and ceftazidime are preferred perioperatively and for 72 h postoperatively (necessitating a 3 day hospitalization), followed at discharge by 10 days of oral dicloxicillin. A single intravenous dose of cefazolin is used at the third-phase surgery. Antibiotics are adjusted appropriately in cases of allergies.

Some children become sleepy or have decreased level of alertness following implantation of 6 or more thalamic test electrodes, presumably due to a “micro-lesion” effect. This effect resolves following electrode removal, but it can interfere with NMU testing if the child is insufficiently cooperative or does not demonstrate dystonia. In these cases methylphenidyate 5–10 mg given orally in the morning during NMU testing significantly improves alertness and responsiveness.

### 2.9. Outcome Measures

Burke–Fahn–Marsden Dystonia Rating Scale (BFMDRS) [23] was performed on video recordings from preoperative and 6 months postoperative visits. Ratings were performed by two trained staff members (A.T. and D.F.) and confirmed independently by the treating neurologist (T.D.S.). Agreement from all 3 raters was required for scoring. The relative timing of the videos could not be blinded since children both are evidently older and almost all children had significant improvement in function visible on the postoperative videos.

Statistical analysis of outcome was perfomed using the R statistical package [24]. The hypothesis that surgery is effective at reducing BFMDRS was tested by separate repeated-measures generalized linear models with BFM score as the dependent variable and time following surgery and subject ID as the dependent variables (calculated using the glm() package in R, with statistical model “BFM ~ factor(subjectID) + Time”). Significance is based on ANOVA (ANOVA () method in R), and the null hypothesis is rejected for *p* < 0.05.

## 3. Results

Table 1 shows the demographics and Burke–Fahn–Marsden Dystonia Rating Scale (BFMDRS) outcomes data for children implanted following testing in the NMU. For comparison, 6 children implanted with 4 electrodes using standard targeting procedures and intraoperative microelectrode recording (MER) are also shown. The comparison cases all had implantation in bilateral GPi and Voa/Vop thalamus, using intraoperative microelectrode recording and stimulation with the patients awakened in the operating room, but without inpatient NMU testing.

Patient NMU3 had only unilateral leads placed to treat hemidystonia. Patient NMU5 had existing leads in bilateral GPi, and additional leads were placed in Vo following NMU testing. For this patient, the pre- and post- surgical ratings were performed before and after implantation in Vo.

We consider an improvement of greater than 5 points on the BFMDRS to be clinically significant. 3 out of 6 children (50%) implanted following recording and stimulation in the operating room (OR) improved. 7 out of 9 children (80%) implanted following recording and stimulation in the Neuromodulation Unit (NMU) improved. The effect of OR-based surgery was not significant (coefficient −10.3, SE (standard error) 4.97, *p* = 0.09), probably because of the small number of subjects. The effect of NMU-based surgery was significant (coefficient −17.1, SE 4.17, *p* = 0.003). The difference in mean improvement between OR-based and NMU-based procedures was not significant (confidence interval −7 to 20, *p* = 0.32).

Evoked potentials consistently showed strong responses to contralateral median nerve stimulation in Vim and (when tested) VPL, with weaker or absent responses in Vo, VA, GPi, and STN. Figure 1 shows a typical example. The latency to the evoked response is typically 12–14 ms on the high impedance channels, and 18–22 ms on the low impedance channels and scalp electrodes, consistent with the expected N20 cortical somatosensory response. The longer latency in the low impedance electrodes most likely reflects low pass filtering due to the tissue-electrode interface and the amplifier filter settings.

Single-unit recordings show low baseline rates of activity, with typical firing rates from 4–10 Hz in both thalamus and pallidum. Firing rates typically increase with voluntary movement, and have a greater level of increase with dystonia. Stretch-evoked responses are seen in VPL (when recorded), and sometimes in GPi (significantly increased response to contralateral elbow or knee stretch was seen in patient NMU3).

### 3.1. Adverse Events

There were no significant perioperative adverse events. Patient NMU5 with pre-existing bilateral GPi leads developed progressive weakness and aphasia following phase 1 surgery with test leads in thalamus. Adtech leads were removed, and subsequently she had worsening cervical dystonia and dysarthria. This required 2 months admission for inpatient rehabilitation. She improved to baseline, and additional permanent leads were implanted in thalamus 6 months after the NMU admission, with improvement in arm function and speech. Examination of the postoperative CT scans showed that the Adtech lead entry points had been placed relatively posterior to avoid her existing permanent GPi leads, and the Adtech leads likely passed through regions of dorsal premotor cortex and parts of the posterior limb of the internal capsule. These regions were avoided in all subsequent cases, and this type of adverse event has not recurred.

Two patients had wound infections, both caused by mechanical trauma to the surgical chest or scalp wound prior to complete healing. In patient NMU1, infection was confirmed with positive cultures necessitating removal of the stimulation system (which had been clinically effective). Return of generalized dystonia following removal of the system led to a return of her preoperative severe disability including failure to thrive and respiratory compromise and she expired 2 months later.

## 4. Discussion

The efficacy of deep brain stimulation will be determined by many factors, among which the target location is likely to be highly important. The procedure we describe here allows more thorough and extensive testing of stimulation efficacy and side effects compared to what can be obtained in the operating room. Children are tested after anesthesia has worn off, and they do not need to be awakened while in a head frame and restrained on the operating table. Despite the apparently increased complexity requiring three procedures instead of one, we expect significantly improved safety because children remain under general endotracheal anesthesia during the procedures with airway protection and controlled physiology. The operating procedures are also shorter and require fewer personnel, because electrophysiological testing does not need to be performed in the operating room. The operating procedure safety is shared with MRI-guided implantation, since children remain anesthetized throughout that procedure as well. However, MRI-guided implantation relies on purely anatomical targeting, and it is not possible to select between targets nor to evaluate for side effects.

Evaluation of side effects is critically important for treatment because they can limit subsequent treatment options. For example, if a child develops paresthesias or muscle spasms at 2.0 V on a particular contact, then it will not be possible to stimulate above that level after permanent implantation, and this may render the contact ineffective. When this is known prior to permanent implantation, lead targets can be moved to avoid internal capsule, more posterior regions of thalamus, or other areas that may contribute to limitation of stimulation parameters.

Prior to using the NMU recording, we had been routinely implanting leads in both GPi and Vo. In 4 of the 9 NMU cases, we ended up selecting a target different from Vo, and thus improved outcomes in these cases can be directly attributed to the new targeting method. It is also important to realize that NMU testing provides a “try-before-you-buy” ability to decide whether or not to proceed with implantation of permanent leads. For example, in one case (not included in Table 1) the benefit in NMU was insufficient and the child was not implanted. In the case of patients with unusual disorders such as the cases of striatal necrosis and folate transporter reported here, there is not sufficient historical data to determine the efficacy or target location for DBS. In these cases, the decision to proceed with implantation can be made on the basis of the child’s own response to stimulation, even when minimal or no historical data are available.

Detailed analysis of the electrophysiological data is in progress and will be reported elsewhere. While there appear to be significant differences in pattern of activity between different children, in general it appears that most or all tested regions increase their firing rates during movement, with greater increases when the movement includes dystonia. For the purposes of targeting, we assume that if firing in a region does not change with the presence or absence of dystonia, then it is unlikely that region is causative for dystonia. The converse is not true, since activity correlating with dystonia could be causative, but could also be associated with, responding to, or compensating for dystonia.

Simultaneous implantation of DBS electrodes in multiple areas has been performed previously, including stimulation of GPi and centromedian nucleus of the thalamus [25,26], GPi and STN [27], or STN and pedunculopontine nucleus (PPN) [28] in Parkinson’s disease. In most cases, stimulation of all 4 leads was superior to stimulation of only two, similar to our results in dystonia. To our knowledge, it appears that the case series reported here is the first to use stereo EEG depth electrodes for pre-implantation testing and target selection for dystonia.

## 5. Conclusions

Deep brain stimulation for secondary dystonia is an important intervention that has the potential for an effect size greater than any currently known medical therapy. Effectiveness depends upon accurate targeting. This small pilot study suggests that the optimal target varies between children, and thus we expect that individualized measurement and targeting will improve outcomes. This small case series demonstrates the feasibility of testing while children are awake and performing functional tasks. Although the method requires three surgical procedures, all procedures are performed with general endotracheal anesthesia which is considerably safer than awakening dystonic children in the operating room for microelectrode recording and side effect testing. Compared with MRI-guided implantation, this procedure allows side effect testing and may improve targeting in secondary dystonia, for which MRI identification of the anatomically most likely target may not adequately predict efficacy. The procedure allows evaluation of potential benefit before implantation of permanent electrodes, and therefore contributes to surgical decision-making. Finally, the procedure provides a wealth of electrophysiological data that can hopefully be used to improve efficacy in the future. Further research and larger case series are needed to confirm these results, to identify all the potential targets for DBS for treatment of dystonia, and to determine whether this procedure may have efficacy for other disorders that are potentially treatable by neurostimulation.

## Figures and Tables

**Table 1:** Demographics and outcomes. Subjects OR1–OR6 had targeting performed in the operating room, while subjects NMU1–NMU9 had targeting performed in the Neuromodulation unit.

Subject	Age	Sex	Diagnosis	Leads	Pre BFM	Post BFM
OR1	18	M	TBI	Rt GPi/Vo	12	10
OR2	14	F	Drowning	Bi GPi/Vo	92	72
OR3	12	F	CP	Bi GPi/Vo	94	88
OR4	15	F	Angelman syndrome	Bi GPi/Vo	108	78
OR5	11	F	Dopamine transporter	Bi GPi/Vo	112	112
OR6	20	M	CP	Bi GPi/Vo	86	82
NMU1	6	F	Idiopathic	Bl GPi/VIM	112	80
NMU2	18	M	CP	Bl GPi/STN	88	88
NMU3	14	M	Idiopathic unilateral	Rt GPi/Vo/VPL	30	24
NMU4	6	F	HUS	Bi GPi/Vo	81	65
NMU5	19	F	Kernicterus	Bi GPi/Vo	84	49
NMU6	14	F	Idiopathic	Bi GPi/VA	64	36
NMU7	20	M	CP	Bi GPi/Vo	113	94
NMU8	7	M	Folate transporter	Bi GPi/Vo	95	91
NMU9	18	F	Stroke	Bi GPi/Vo	28	13

TBI = traumatic brain injury, CP = cerebral palsy, HUS = hemolytic-uremic syndrome.
